# Supplementary material for: Associations between β-blockers and psychiatric and behavioural outcomes: A population-based cohort study of 1.4 million individuals in Sweden
Source: PLoS Med. 2023 Jan 31;20(1):e1004164. doi: 10.1371/journal.pmed.1004164 (PMC9888684; doi:10.1371/journal.pmed.1004164)
Supplement: S1 Checklist — (PDF) [file pmed.1004164.s001.pdf]

STROBE Statement—Checklist of items that should be included in reports of ***cohort studies***

	Item No	Recommendation	Page No
**Title and abstract**	1	(*a*) Indicate the study's design with a commonly used term in the title or the abstract<br>"Associations between β-blockers and psychiatric and behavioural outcomes – a population-based study of 1.4 million individuals"<br>(*b*) Provide in the abstract an informative and balanced summary of what was done and what was found	(a) Title page<br><br>(b) p. 3-4
**Introduction**			
Background/rationale	2	Explain the scientific background and rationale for the investigation being reported	Introduction ¶ 1-6
Objectives	3	State specific objectives, including any prespecified hypotheses<br>"We examined associations between β-blocker use and psychiatric and behavioural outcomes, including hospitalisations for psychiatric disorders, suicidal behaviour and deaths from suicide, and charges of violent crime, by applying a within-individual design (i.e. we compared individuals to themselves during medication and non-medication periods (54)) in a population-based cohort of 1.4 million β-blocker users who were followed for 8 years."	Introduction ¶ 7
**Methods**			
Study design	4	Present key elements of study design early in the paper<br>"We conducted a population-based longitudinal cohort study using Swedish nationwide registers linked through each person's unique identification number (55). Registers included the Total Population Register (for information on age, sex, and migration), the Swedish Prescribed Drug Register (for information on dispensed medications), the Swedish Patient Register (for information on diagnoses, hospitalisations, and treatment of suicidal behaviour), the Cause of Death Register (for information on death by suicide and other causes), the Register of Persons Suspected of Offences (for information on charges for violent and non-violent crime), the Longitudinal Integrated Database for Health Insurance and Labour Market Studies (LISA; for information on civil status and source of income), and the Prison and Probation Services Register (for information on periods in prison) (55-60). For more details on the registers, see S1 Text, p. 2. We applied a within-individual design (61) that inherently adjusts for all stable confounders, i.e. factors that do not change during the study period (e.g. genetics and health history), and more fully adjusts for stable factors associated with confounding by indication. "	Methods - Design ¶ 1
Setting	5	Describe the setting, locations, and relevant dates, including periods of recruitment, exposure, follow-up, and data collection<br>"We identified all individuals with dispensed β-blockers (i.e. filled-in prescriptions) in the Swedish population aged 15 and older (i.e. the age of criminal responsibility). Data on medication exposure in the Prescribed Drug Register was available from July 1, 2005, however, all information on each collected prescription was not complete in 2005 (62). The study period therefore started in January 1, 2006 and ended in December 31, 2013 (the last available date for register linkage)."	Methods - Participants and setting ¶ 1
Participants	6	(*a*) Give the eligibility criteria, and the sources and methods of selection of participants. Describe methods of follow-up<br>"Initially, we identified 1,628,655 individuals who had been dispensed a β-blocker between 2006 and 2013. We excluded individuals with other treatment patterns (S1 Fig); such as individuals who collected a single prescription (n=134,336), individuals PRN instructions (n=64,822), individuals under age 15, i.e. under the age of criminal responsibility in Sweden (n=2,729), and individuals with irregularly collected prescriptions, that is, where new prescriptions were collected more than six months after the previous one (n=26,002). The final cohort included 1,400,766 individuals."	(a) Methods - Medications ¶ 2<br><br>(b) n/a (cohort study)

		(*b*) For matched studies, give matching criteria and number of exposed and unexposed	
Variables	7	Clearly define all outcomes, exposures, predictors, potential confounders, and effect modifiers. Give diagnostic criteria, if applicable	p. Methods ¶ 3-7 + S1 Text
Data sources/ measurement	8*	For each variable of interest, give sources of data and details of methods of assessment (measurement). Describe comparability of assessment methods if there is more than one group	p. Methods ¶ 3-7 + S1 Text
Bias	9	Describe any efforts to address potential sources of bias<br>In sensitivity analyses – alternative exposures and outcomes, and sensitivity analyses – alternative samples	Methods – Statistical analyses ¶ 1-8
Study size	10	Explain how the study size was arrived at<br>"Initially, we identified 1,628,655 individuals who had been dispensed a β-blocker between 2006 and 2013. We excluded individuals with other treatment patterns (S1 Fig); such as individuals who collected a single prescription (n=134,336), individuals PRN instructions (n=64,822), individuals under age 15, i.e. under the age of criminal responsibility in Sweden (n=2,729), and individuals with irregularly collected prescriptions, that is, where new prescriptions were collected more than six months after the previous one (n=26,002). The final cohort included 1,400,766 individuals."	Methods - Medications ¶ 2
Quantitative variables	11	Explain how quantitative variables were handled in the analyses. If applicable, describe which groupings were chosen and why	Methods – Statistical analyses ¶ 1-8
Statistical methods	12	(*a*) Describe all statistical methods, including those used to control for confounding	(a) Methods – Statistical analyses ¶ 1-5
		(*b*) Describe any methods used to examine subgroups and interactions<br>Described in: sensitivity analyses – alternative exposures and outcomes, and sensitivity analyses – alternative samples, and sensitivity analyses – posthoc analyses	(b) Methods – Statistical analyses ¶ 6-8
		(*c*) Explain how missing data were addressed	(c) n/a (register data with nationwide coverage)
		(*d*) If applicable, explain how loss to follow-up was addressed<br>"All individuals in the cohort were followed from the start of the study period (January 1, 2006), or the date of immigration to Sweden, and were censored at death, emigration, or the end of study period (December 31, 2013), whichever occurred first."	(d) Methods – Statistical analyses ¶ 1
		(*e*) Describe any sensitivity analyses<br>Described in: sensitivity analyses – alternative exposures and outcomes, and sensitivity analyses – alternative samples, and sensitivity analyses – posthoc analyses	(e) Methods – Statistical analyses ¶ 6-8
**Results**			
Participants	13*	(a) Report numbers of individuals at each stage of study—eg numbers potentially eligible, examined for eligibility, confirmed eligible, included in the study, completing follow-up, and analysed<br>"We identified 1,628,655 individuals who had been prescribed β-blockers during the study period between 2006 and 2013. After exclusions due to irregular medication use and age (S1 Fig), the final cohort included 1,400,766 individuals (15.7% of the total population of Sweden aged 15 years or older during the study period [n=8,945,456])."	(a) Results ¶ 1
		(b) Give reasons for non-participation at each stage<br>See above	(b) Results ¶ 1
		(c) Consider use of a flow diagram	(c) S1 Fig

		S1 Fig	
Descriptive data	14*	(a) Give characteristics of study participants (eg demographic, clinical, social) and information on exposures and potential confounders<br>(b) Indicate number of participants with missing data for each variable of interest<br>(c) Summarise follow-up time (eg, average and total amount)	(a) Results ¶ 1 + Table 1<br>(b) n/a<br>(c) S1 Table
Outcome data	15*	Report numbers of outcome events or summary measures over time	Tables 1-3, S2 Table

Main results	16	(*a*) Give unadjusted estimates and, if applicable, confounder-adjusted estimates and their precision (eg, 95% confidence interval). Make clear which confounders were adjusted for and why they were included<br>(*b*) Report category boundaries when continuous variables were categorized<br>(*c*) If relevant, consider translating estimates of relative risk into absolute risk for a meaningful time period	(a) Figs 1-3, Tables 2-3, S2 Fig, S4 Table<br>(b) n/a<br>(c) Table 1
Other analyses	17	Report other analyses done—eg analyses of subgroups and interactions, and sensitivity analyses	Tables 2-3, S2 Fig
**Discussion**			
Key results	18	Summarise key results with reference to study objectives<br>"In this population-based cohort of 1.4 million persons in Sweden who had been treated with β-blockers between 2006 and 2013, we used a within-individual design that accounted for background factors associated with confounding by indication. We found some heterogeneity in the direction of associations of β-blockers with the psychiatric and behavioural outcomes investigated; notably, we found that periods on β-blocker treatment were associated with decreased psychiatric hospitalisation hazards (HR: 0.92, 95% CI: 0.91-0.93, p<.001) as compared to periods off treatment. In addition, there was a 13% (HR: 0.87, 95% CI: 0.81-0.93, p<.001) lower risk of being charged with a violent crime by the police or prosecution services during β-blocker treatment. In contrast, there was a small increased association with treatment for suicidal behaviour and suicide mortality (HR: 1.08, 95% CI: 1.02-1.15, p=.012; with 0.7% of the cohort experiencing this outcome during the study period) during β-blocker treatment. We carried out several sensitivity analyses to test the robustness of results, and reduced associations with violent crime during β-blocker treatment periods were consistent. However, associations with reduced psychiatric hospitalisations and increased suicidal behaviour during β-blocker treatment shown in the principal analyses were not consistent across all sensitivity analyses, suggesting that these findings could be partially confounded."	Discussion ¶ 1
Limitations	19	Discuss limitations of the study, taking into account sources of potential bias or imprecision. Discuss both direction and magnitude of any potential bias	Discussion ¶ 2-9
Interpretation	20	Give a cautious overall interpretation of results considering objectives, limitations, multiplicity of analyses, results from similar studies, and other relevant evidence	Discussion ¶ 2-9
Generalisability	21	Discuss the generalisability (external validity) of the study results	Discussion ¶ 9
**Other information**			
Funding	22	Give the source of funding and the role of the funders for the present study and, if applicable, for the original study on which the present article is based<br>"This study was supported by the Wellcome Trust (No 202836/Z/16/Z): https://wellcome.org/grant-funding (SF), the Swedish Research Council for Health Working Life and Welfare (2015-0028): https://forte.se/en/ (PL and HL), the American Foundation for Suicide Prevention (DIG-1-037-19): https://afsp.org/research-grant-information (BMD), and Karolinska Institutet Funds (2016fobi50581): https://staff.ki.se/ki-foundations-funds-list-of-grants (YM). The funders had no role in study design, data collection and analysis, decision to publish, or preparation of the manuscript"	In submission statement

*Give information separately for exposed and unexposed groups.

Note: An Explanation and Elaboration article discusses each checklist item and gives methodological background and published examples of transparent reporting. The STROBE checklist is best used in conjunction with this article (freely available on the Web sites of PLoS Medicine at http://www.plosmedicine.org/, Annals of Internal Medicine at http://www.annals.org/, and Epidemiology at http://www.epidem.com/). Information on the STROBE Initiative is available at http://www.strobe-statement.org.
