# Supplementary figures and images for: Associations between β-blockers and psychiatric and behavioural outcomes: A population-based cohort study of 1.4 million individuals in Sweden
Source: PLoS Med. 2023 Jan 31;20(1):e1004164. doi: 10.1371/journal.pmed.1004164 (PMC9888684; doi:10.1371/journal.pmed.1004164)

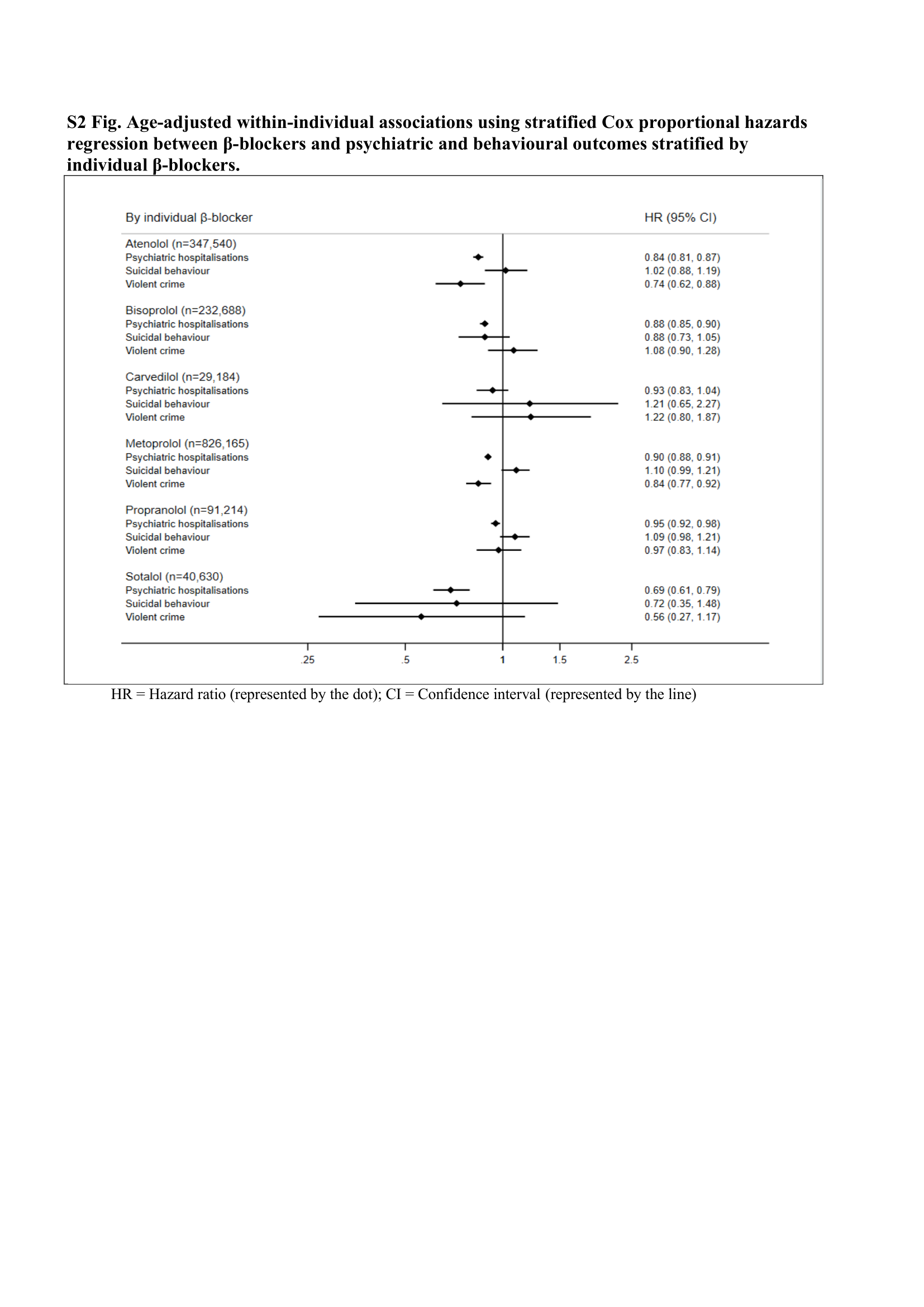

Supplement: S2 Fig — (TIFF) [file pmed.1004164.s003.tiff]
