# Supplementary material for: Associations between β-blockers and psychiatric and behavioural outcomes: A population-based cohort study of 1.4 million individuals in Sweden
Source: PLoS Med. 2023 Jan 31;20(1):e1004164. doi: 10.1371/journal.pmed.1004164 (PMC9888684; doi:10.1371/journal.pmed.1004164)
Supplement: S1 Text — (DOCX) [file pmed.1004164.s008.docx]

**Supplementary material to the manuscript**

Associations between β-blockers and psychiatric and behavioural outcomes:

A population-based cohort study of 1.4 million individuals in Sweden

Yasmina Molero, Sam Kaddoura, Ralf Kuja-Halkola, Henrik Larsson, Paul Lichtenstein, Brian M. D’Onofrio, and Seena Fazel

**Contents**

Supplementary methods ...…………………………………………………………………………………………2

Registers ...…………………………………………………………………………………………………………2

β-blockers ….………………………………………………………………………………………………………2

Psychiatric and behavioural outcomes ……....……………………………………………………………………..3

Other medications ...……………………………………………………………………………………………......3

Negative controls ...…………………………………………………………………………………………….......4

Diseases of the circulatory system …………......…………………………………………………………………..5

Indications for treatment ......…………………………………………………………………………………….....5

Stratified Cox proportional hazards regression ...…………………………………………………………………..6

References ...……………………………………………………………………………………………………..... 7

**Supplementary Methods**

**Registers**

Registers included the Total Population Register, the Swedish Prescribed Drug Register, the Swedish Patient Register, the Cause of Death Register, the Register of Persons Suspected of Offences, and the Prison and Probation Services Register [1-7]. The Total Population Register contains information on birth, sex, and migration for the entire Swedish Population. The Swedish Prescribed Drug Register includes information on all prescriptions that are dispensed from all pharmacies in Sweden, and has less than 0.3% missing information. The Swedish Patient Register includes all admissions to all hospitals, as well as all outpatient contacts with specialized secondary care in Sweden. The primary diagnosis is listed in 99% of all hospital discharges, and the positive predictive value of medical diagnoses in this register is around 85-95%. In a previous register-based study, depression diagnoses were validated by comparing concordance rates with another clinical register that was based on multidisciplinary inpatient assessments, to make diagnoses as a gold standard. Results showed fair to moderate agreement (κ of 0.32; 88% full agreement) [8]. Missing data in The Swedish Patient Register is around 1% for inpatient treatment, and around 3% for outpatient treatment. The Cause of Death Register is a register of all deaths in Sweden, where the underlying cause is specified in 96% of the cases. The Register of Persons Suspected of Offences includes all individuals who are charged with a crime after a completed investigation by police, prosecution services, or customs authority. The Longitudinal Integrated Database for Health Insurance and Labour Market Studies (LISA) covers the adult Swedish population aged ≥16 years and contains sociodemographic data such as family, employment, benefits, and education for each calendar year. The Prison and Probation Services Register includes information on all prison sentences, including the start and end date of each prison sentences.

**β-blockers**

Information on beta-adrenergic blocking agents, i.e. β-blockers, was collected from the Swedish Prescribed Drug Register [4], and included atenolol (Anatomical Therapeutic Chemical [ATC] classification system: C07AB03), bisoprolol (ATC: C07AB07), carvedilol (ATC: C07AG02), labetalol (ATC: C07AG01), metoprolol (ATC: C07AB02), pindolol (ATC: C07AA03), propranolol (ATC: C07AA05), and sotalol (ATC: C07AA07). β-blockers were first analysed as a whole class, and then separately: 1) by selectivity (β1 selective β-blockers: atenolol, bisoprolol, and metoprolol; non-selective β-blockers: carvedilol, labetalol, pindolol, propranolol, and sotalol); 2) by solubility (hydrophilic β-blockers: atenolol and sotalol; lipophilic β-blockers; bisoprolol, carvedilol, labetalol, metoprolol, pindolol, and propranolol), and; 3) by individual β-blockers (atenolol, bisoprolol, carvedilol, metoprolol, propranolol, and sotalol - treatment periods with labetalol and pindolol did not include enough outcome events to allow for separate analyses).

**Psychiatric and behavioural outcomes**

Information on all psychiatric outcomes in the main analyses was collected from the Swedish Patient Register [3]. In our study, we included only psychiatric diagnoses received during inpatient care in the main analyses. In sensitivity analyses, we included only psychiatric diagnoses made in specialised open care during unplanned visits. For suicidal behaviour, we included all unplanned hospitalisations and open care visits. This included all visits to physicians – both at hospitals, emergency rooms, and specialist outpatient care – that were not made by prior appointment. Diagnoses received during planned visits (i.e. follow-ups and referrals) were excluded. Although this is a more conservative approach, this measure was used to avoid outcome overestimation, as the diagnosis that is the reason for treatment initiation may also be coded during follow-ups and referrals regardless of current symptoms. In addition, information on deaths from suicide and other causes was collected from the Cause of Death Register.

We also tested for associations with charges of violent and non-violent crimes, extracted from the Register of Persons Suspected of Offences [6]. We used crime charges, rather than convictions, as some charges may dropped by the prosecution before going to court, such as when an individual has committed several crimes [9]. The date when the crime was committed (rather than charged) was used. If more than one crime was committed on the same day, only the first crime was counted (for violent and non-violent crimes, respectively). Violent crimes were defined as crimes against people in the Swedish penal code [10], and included attempted, completed, and aggravated forms of murder, manslaughter, unlawful threats, harassment, robbery, arson, assault, assault on an official, kidnapping, stalking, coercion, and all sexual offences except purchase of sexual services. Non-violent crimes were defined as all other crimes.

**Other medications**

In sensitivity analyses, we excluded individuals who had collected at least one prescription for medications that have been linked to psychiatric and behavioural outcomes; antidepressant medications (ATC: N06A) [11,12], antipsychotics (ATC: N05A) [13], benzodiazepines (ATC: N03AE, N05BA, N05CD, N05CF) [14], calcium channel blockers (ATC: C08)[15], renin-angiotensin system acting agents (ATC: C09) [16], or statins (ATC: C10AA) [17], all extracted from the Swedish Prescribed Drug Register. In further sensitivity analyses, we excluded individuals with polypharmacy, that is, those who had collected prescriptions for five or more different medication classes, including β-blockers, during the same calendar year. Medication classes included medications for the alimentary tract and metabolism (ATC: A01, A02, A07, A10), cardiovascular medications (ATC: C01-C03, C05, C07-C10), dermatologicals (ATC: D04-D07, D11), genito-urinary system and sex hormones (ATC: G02-G04), systemic hormonal preparations (ATC: H01-H05), anti-infectives (ATC: J01, J02, J04, J05), antineoplastic and immunomodulating agents (ATC: L01-L04), musculo-skeletal system medications (ATC: M01), psychotropics (ATC: N), anti-parasitics (ATC: P01, P02), respiratory system agents (ATC: R01, R03, R05, R06), and sensory organ medications (ATC: S01).

**Negative controls**

We used two medications - angiotensin-converting-enzyme (ACE) inhibitors (ATC: C09AA) and antihistamines for systemic use (ATC: R06A; excluding phenothiazine derivatives [ATC: R06AD], as these are used clinically as mild sedatives to treat anxiety) - as negative controls to test for non-specific treatment effects. This choice was determined on the basis of theoretical reasons; ACE inhibitors are prescribed for similar indications and in similar settings as β-blockers, and would thus likely capture similar non-specific treatment effects, such as increased supervision and healthcare contacts. Antihistamines, on the other hand, are prescribed for other indications and in other settings than β-blockers, and would thus likely capture non-specific treatment effects not related to cardiac treatment (since β-blockers can be prescribed for non-cardiac indications). In these analyses, the negative control medications were used as an independent exposure in the β-blockers cohort, and treatment periods were defined in the same manner as β-blocker treatment periods (i.e. at least two dispenses within six months). Because ACE inhibitors and β-blockers are often co-prescribed, we adjusted for the effect of concurrent β-blocker use in the ACE inhibitors analyses. Information was extracted from the Swedish Prescribed Drug Register.

**Diseases of the circulatory system**

Diagnosed diseases of the circulatory system included acute rheumatic fever (ICD-10: I00-I02), chronic rheumatic heart diseases (ICD-10: I05-I09), hyperintensive diseases (ICD-10: I10-I15), ischaemic heart diseases (ICD-10: I20-I25), pulmonary heart disease and diseases of pulmonary circulation (ICD-10: I26-I28), other forms of heart disease (ICD-10: I30-I52), cerebrovascular diseases (ICD-10: I60-I69), diseases of arteries, arterioles and capillaries (ICD-10: I70-I79), diseases of veins, lymphatic vessels and lymph nodes (ICD-10: I80-I89), and other and unspecified disorders of the circulatory system (ICD-10: I95-I99).

**Indications for treatment**

In further analyses, each prescription was stratified on the indication for treatment, i.e. the reason for prescribing the medication as stated in the prescription text written by the prescribing doctor. We categorised indications into three hierarchical and mutually exclusive categories: 1) psychiatric or behavioural indications; 2) cardiac indications, and; 3) other or unspecified indications. Consequently, individuals with at least one prescription with a psychiatric or behavioural indication were regarded as having an indication within this category, regardless of additional indications. Individuals with a cardiac indication, but no psychiatric or behavioural indication, were regarded as having an indication within this category. Individuals with other or unspecified indications, but no psychiatric, behavioural or cardiac indication were regarded as having an indication within this category. To identify and classify indications, we first extracted a subset of 15,373 random entries of prescription texts from the Prescribed Drug Register. Each string (in Swedish) was then screened and manually annotated with the corresponding indication (e.g. heart failure was annotated as cardiac condition). Based on this screening, we compiled a list of multiple ways in which each indication could be described in the prescription text (see S3 Table for more details). After compiling the initial list, we extracted another 30,000 random entries of prescription texts for further screening of indications. Following several iterations of testing in the entire subset of randomly extracted prescription texts (a total screening input of 45,373 prescription text entries), we identified a number of variations for each indication (S3 Table). We then compiled a set of identifiers used to flag indications in the entire β-blocker cohort. A proportion of prescription text entries were blank, as prescription texts would not always be reiterated after the first prescription. For blank prescription texts, we identified previous prescription text entries, and matched them with the corresponding prescription information.

**Stratified Cox proportional hazards regression**

We used stratified Cox proportional hazards regression to examine associations between medications and outcomes [18]. In this model, each individual is entered as a separate stratum in the analysis and serves as his/her own control. The obtained hazard ratio is thus adjusted for (i.e. stratified by) all time-invariant confounders within each individual. In the within-individual design, all individuals contribute; either directly to the estimate of medication exposure on the outcome (i.e. those who change from on treatment to off treatment or vice versa, and also experience an outcome during either a medication or non-medication period), or indirectly (i.e. through the association of other individual-level covariates adjusted for in the model, such as age, if there is within-individual variation in at least one of these covariates). To estimate cause-specific hazard ratios, we treated the competing event of death as a censoring event, rather than fitting competing risks models. Using a competing risk analysis, such as the Fine and Gray model, would estimate the subdistribution hazard ratio. Such a hazard ratio would not only capture any potential effect of exposure (β-blockers) on the outcome of interest (psychiatric hospitalisations, suicidal behavior, or violent crime) but would also be influenced by any potential effect of the exposure on the competing event (death) [19]. In the current study, this was not appropriate, wherefore we opted to estimate the cause-specific hazard ratio.

**References**

1. Brooke HL, Talback M, Hornblad J, Johansson LA, Ludvigsson JF, Druid H, et al. The Swedish cause of death register. Eur J Epidemiol. 2017;32(9):765-73.

2. Ludvigsson JF, Almqvist C, Bonamy AK, Ljung R, Michaëlsson K, Neovius M, et al. Registers of the Swedish total population and their use in medical research. Eur J Epidemiol. 2016;31(2):125-36.

3. Ludvigsson JF, Andersson E, Ekbom A, Feychting M, Kim JL, Reuterwall C, et al. External review and validation of the Swedish national inpatient register. BMC Public Health. 2011;11:450.

4. Wettermark B, Hammar N, Fored CM, Leimanis A, Otterblad Olausson P, Bergman U, et al. The new Swedish prescribed drug register--opportunities for pharmacoepidemiological research and experience from the first six months. Pharmacoepidemiol Drug Saf. 2007;16(7):726-35.

5. Ludvigsson JF, Otterblad-Olausson P, Pettersson BU, Ekbom A. The Swedish personal identity number: possibilities and pitfalls in healthcare and medical research. Eur J Epidemiol. 2009;24(11):659-67.

6. Brottsförebyggande rådet. Kriminalstatistik 2018. Personer lagförda för brott. Slutlig statistik. Stockholm: Brottsförebyggande rådet URN: NBN: SE: BRA-835; 2019. p. 1-64.

7. Ludvigsson JF, Svedberg P, Olen O, Bruze G, Neovius M. The longitudinal integrated database for health insurance and labour market studies (LISA) and its use in medical research. Eur J Epidemiol. 2019;34(4):423-37.

8. Fazel S, Wolf A, Chang Z, Larsson H, Goodwin GM, Lichtenstein P. Depression and violence: a Swedish population study. Lancet Psychiatry. 2015;2(3):224-32.

9. The Swedish Government Official Reports 2006:30 [Statens Offentliga Utredningar 2006:30]. Is justice fair? Ten perspectives on discrimination of ethnic and religious minorities in the judicial system [Är rättvisan rättvis? Tio perspektiv på diskriminering av etniska och religiösa minoriteter inom rättssystemet] Stockholm: SOU 2006:30 ISSN 0375-250X; 2006.

10. Fazel S, Långström N, Hjern A, Grann M, Lichtenstein P. Schizophrenia, substance abuse, and violent crime. JAMA. 2009;301(19):2016-23.

11. Molero Y, Lichtenstein P, Zetterqvist J, Gumpert CH, Fazel S. Selective serotonin reuptake inhibitors and violent crime: a cohort study. PLoS Med. 2015;12(9):e1001875-e.

12. Lagerberg T, Fazel S, Molero Y, Franko MA, Chen Q, Hellner C, et al. Associations between selective serotonin reuptake inhibitors and violent crime in adolescents, young, and older adults – a Swedish register-based study. Eur Neuropsychopharmacol. 2020;36:1-9.

13. Fazel S, Zetterqvist J, Larsson H, Långström N, Lichtenstein P. Antipsychotics, mood stabilisers, and risk of violent crime. Lancet. 2014;384(9949):1206-14.

14. Albrecht B, Staiger PK, Hall K, Miller P, Best D, Lubman DI. Benzodiazepine use and aggressive behaviour: a systematic review. Aust N Z J Psychiatry 2014;48(12):1096-114.

15. Cipriani A, Saunders K, Attenburrow MJ, Stefaniak J, Panchal P, Stockton S, et al. A systematic review of calcium channel antagonists in bipolar disorder and some considerations for their future development. Mol Psychiatry. 2016;21(10):1324-32.

16. Vian J, Pereira C, Chavarria V, Köhler C, Stubbs B, Quevedo J, et al. The renin-angiotensin system: a possible new target for depression. BMC Med. 2017;15(1):144.

17. Molero Y, Cipriani A, Larsson H, Lichtenstein P, D'Onofrio BM, Fazel S. Associations between statin use and suicidality, depression, anxiety, and seizures: a Swedish total-population cohort study. Lancet Psychiatry. 2020;7(11):982-90.

18. Lichtenstein P, Halldner L, Zetterqvist J, Sjolander A, Serlachius E, Fazel S, et al. Medication for attention deficit-hyperactivity disorder and criminality. N Engl J Med. 2012;367(21):2006-14.

19. Bhaskaran K, Rachet B, Evans S, Smeeth L. Re: Helene Hartvedt Grytli, Morten Wang Fagerland, Sophie D. Fosså, Kristin Austlid Taskén. Association between use of β-blockers and prostate cancer-specific survival: a cohort study of 3561 prostate cancer patients with high-risk or metastatic disease. Eur Urol. In press. <http://dx.doi.org/10.1016/j.eururo.2013.01.007>.: beta-blockers and prostate cancer survival--interpretation of competing risks models. Eur Urol. 2013;64(4):e86-7.
