# Supplementary material for: Associations between β-blockers and psychiatric and behavioural outcomes: A population-based cohort study of 1.4 million individuals in Sweden
Source: PLoS Med. 2023 Jan 31;20(1):e1004164. doi: 10.1371/journal.pmed.1004164 (PMC9888684; doi:10.1371/journal.pmed.1004164)
Supplement: Attachment — Submitted filename: Reviewer comments_Molero_PLoSMed.pdf [file pmed.1004164.s009.pdf]

Requests from the editors:

GENERAL

Please address all reviewer and editor comments detailed below

Please remove the Header from each page

Response:

We have removed the header.

Please ensure that the study is reported according to the STROBE guideline, and include the completed STROBE checklist as Supporting Information. Please add the following statement, or similar, to the Methods: "This study is reported as per the Strengthening the Reporting of Observational Studies in Epidemiology (STROBE) guideline (S1 Checklist)." The STROBE guideline can be found here: http://www.equator-network.org/reporting-guidelines/strobe/

When completing the checklist, please use section and paragraph numbers, rather than page numbers.

Response:

We have updated the STROBE checklist using section and paragraph numbers, and also added the suggested statement to the Methods, in the next to last paragraph.

Please remove the data availability statement and competing interests statement from the end of the manuscript and include only in the submission form

Response:

We have removed both.

TITLE

Please revise your title according to PLOS Medicine's style. Your title must be nondeclarative and not a question. It should begin with main concept if possible. "Effect of" should be used only if causality can be inferred, i.e., for an RCT. Please place the study design ("A randomized controlled trial," "A retrospective study," "A modelling

study," etc.) in the subtitle (ie, after a colon).

Response:
We have changed our title to the following: "Associations between β-blockers and psychiatric and behavioural outcomes: A population-based cohort study of 1.4 million individuals in Sweden".

ABSTRACT
*** Please structure your abstract using the PLOS Medicine headings (Background, Methods and Findings, Conclusions).**
*** Please combine the Methods and Findings sections into one section, "Methods and findings".**

Response:
Abstract has been structured as instructed, and Methods and Findings sections have been combined.

Abstract Methods and Findings:
*** Please ensure that all numbers presented in the abstract are present and identical to numbers presented in the main manuscript text.**
*** Please include the study design, population and setting, number of participants, years during which the study took place, length of follow up, and main outcome measures.**
*** Please quantify the main results p-values as well as with 95% CIs. To improve reader accessibility I would suggest removal of the "=" symbol and present your data as follows: "(HR: 0.87, 95% CI: 0.81- 65 0.93, p<0.01)" for example**
*** Please include the important dependent variables that are adjusted for in the analyses.**
*** In the last sentence of the Abstract Methods and Findings section, please describe the main limitation(s) of the study's methodology (as opposed to the limitations of the observational nature of the study).**

Response:
We have ensured that all numbers presented in the Abstract are correct. The Abstract now includes the study design, population and setting, number of participants, years during which

the study took place, length of follow up, and main outcome measures. We also added p-values and removed the "=" symbol (in the Results section too). Furthermore, we included all important variables that were adjusted for, and we describe the main limitation of the study's methodology.

Abstract (underlined added):

Methods and findings

We conducted a population-based longitudinal cohort study using Swedish nationwide high-quality healthcare, mortality, and crime registers. We included 1,400,766 individuals aged 15 years or older who had collected β-blocker prescriptions and followed them for 8 years between 2006 and 2013. We linked register data on dispensed β-blocker prescriptions with main outcomes; hospitalisations for psychiatric disorders (not including self-injurious behaviour or suicide attempts), suicidal behaviour (including deaths from suicide), and charges of violent crime. We applied within-individual Cox proportional hazards regression to compare periods on treatment with periods off treatment within each individual in order to reduce possible confounding by indication, as this model inherently adjusts for all stable confounders (e.g. genetics and health history). We also adjusted for age as a time-varying covariate. In further analyses, we adjusted by stated indications, prevalent users, cardiac severity, psychiatric and crime history, individual β-blockers, β-blocker selectivity and solubility, and use of other medications. In the cohort, 86.8% (n=1,215,247) were 50 years and over, and 52.2% (n=731,322) were women. During the study period, 6.9% (n=96,801) of the β-blocker users were hospitalised for a psychiatric disorder, 0.7% (n=9,960) presented with suicidal behaviour, and 0.7% (n=9,405) were charged with a violent crime. There was heterogeneity in the direction of results; within-individual analyses showed that periods of β-blocker treatment were associated with reduced hazards of psychiatric hospitalisations (hazard ratio [HR]: 0.92, 95% confidence interval [CI]: 0.91-0.93, p<.001) and charges of violent crime (HR: 0.87, 95% CI: 0.81-0.93, p<.001), and increased hazards of suicidal behaviour (HR: 1.08, 95% CI: 1.02-1.15, p=.012). After stratifying by diagnosis, reduced associations with psychiatric hospitalisations during β-blocker treatment were mainly driven by lower hospitalisation rates due to depressive (HR: 0.92, 95% CI: 0.89-0.96, p<.001) and psychotic disorders (HR: 0.89, 95% CI: 0.85-0.93, p<.001). Reduced associations with violent charges remained in most sensitivity analyses, while associations with psychiatric hospitalisations and suicidal behaviour were inconsistent. Limitations include that the within-individual model does not account for confounders that could change during treatment, unless measured and

adjusted for in the model.

AUTHOR SUMMARY

At this stage, we ask that you include a short, non-technical Author Summary of your research to make findings accessible to a wide audience that includes both scientists and non-scientists. The Author Summary should immediately follow the Abstract in your revised manuscript. This text is subject to editorial change and should be distinct from the scientific abstract. Please see our author guidelines for more information: https://journals.plos.org/plosmedicine/s/revising-your-manuscript#loc-author-summary

Response:

We have added the author summary after the Abstract:

Why Was This Study Done?

- β-blockers are primarily cardiac medications that are widely used for treating anxiety, and are also suggested for the management of clinical depression and aggression, although research on efficacy is conflicting and limited by small samples and methodological problems.
- β-blockers have been linked to an increased risk of suicidal behaviour, but findings are inconclusive.
- More evidence using large samples and appropriate designs is needed on real-world effects on mental health and behavioural outcomes in people taking β-blockers.

What Did the Researchers Do and Find?

- We examined a population-based cohort of 1,400,766 persons in Sweden who had been treated with β-blockers using a within-individual design that accounted for background factors that may confound associations.
- Periods on β-blocker treatment were associated with an 8% lower risk of being hospitalised due to a psychiatric disorder, a 13% lower risk of being charged with a violent crime by the police, and an 8% increased risk of being treated for suicidal behaviour or suicide mortality.
- Reduced associations with violent charges were consistent across sensitivity analyses, while associations with suicidal behaviour and psychiatric hospitalisations varied by specific psychiatric diagnoses, past psychiatric problems, and cardiac severity.

What Do These Findings Mean?

• The widespread use of β-blockers to manage anxiety is not supported in this real-world study that examined new presentations of anxiety in secondary care.

• Studies using other designs (e.g. randomised controlled trials) are needed to better understand the role of β-blockers in the management of aggression and violence.

• If findings on violence are triangulated using other designs, β-blockers could be considered to manage aggression and hostility in individuals with psychiatric conditions.

INTRODUCTION

Please remove the sub-heading "Aims" line 138 and conclude the introduction with a clear description of the study question/hypotheses.

Response:

We have removed the sub-heading "Aims", and also revised the study question as follows: We examined associations between β-blocker use and psychiatric and behavioural outcomes, including hospitalisations for psychiatric disorders, suicidal behaviour and deaths from suicide, and charges of violent crime, by applying a within-individual design (i.e. we compared individuals to themselves during medication and non-medication periods (54)) in a population-based cohort of 1.4 million β-blocker users who were followed for 8 years.

METHODS and RESULTS

In the manuscript text, please ensure you have included the following

(1) the specific hypotheses you intended to test,

(2) the analytical methods by which you planned to test them,

(3) the analyses you actually performed, and

(4) when reported analyses differ from those that were planned, transparent explanations for differences that affect the reliability of the study's results. If a reported analysis was performed based on an interesting but unanticipated pattern in the data, please be clear that the analysis was data-driven.

Response:

Our specific hypothesis, the methods, and analyses are included in the Statistical analyses, 2nd paragraph (underlined added):

Our null hypothesis was that no associations would be demonstrated between β-blockers and psychiatric hospitalisations, suicidal behaviour, and violent crime. A within-individual design – using stratified Cox proportional hazards regression - was applied to examine associations.

We have also clarified which analyses were data-driven. In the Sensitivity analyses – alternative exposures and outcomes, 1st paragraph (underlined added):
We carried out several data-driven sensitivity analyses with alternative exposures and secondary outcomes to test the robustness of the results.

In the Sensitivity analyses – alternative samples, 1st paragraph (underlined added):
We carried out further data-driven sensitivity analyses with alternative samples to test the robustness of the results.

Did your study have a prospective protocol or analysis plan? Please state this (either way) early in the Methods section.
a) If a prospective analysis plan (from your funding proposal, IRB or other ethics committee submission, study protocol, or other planning document written before analyzing the data) was used in designing the study, please include the relevant prospectively written document with your revised manuscript as a Supporting Information file to be published alongside your study, and cite it in the Methods section. A legend for this file should be included at the end of your manuscript.
b) If no such document exists, please make sure that the Methods section transparently describes when analyses were planned, and when/why any data-driven changes to analyses took place.
c) In either case, changes in the analysis-- including those made in response to peer review comments-- should be identified as such in the Methods section of the paper, with rationale.

Response:
We have identified which analyses where data-driven (please see our response above). Each of the sensitivity analyses also include a motivation as to why they were performed. We have also clarified which analyses were added during the review process by adding a new paragraph: Sensitivity analyses – posthoc analyses. Here we write:

We carried out several posthoc sensitivity analyses to further test the robustness of results. We further examined non-specific treatment effects by using a different negative control medication - angiotensin-converting-enzyme (ACE) inhibitors (ATC: C09AA) - as an independent exposure in the β-blocker cohort (see S1 Text p. 4 for details). Furthermore, we carried out analyses where we excluded individuals who had been prescribed benzodiazepines (ATC: N03AE, N05BA, N05CD, N05CF) to address the confounding effects of concurrent benzodiazepine use on psychiatric and behavioural outcomes. We also controlled for the confounding effects of polypharmacy by excluding individuals who had been prescribed five or more different medication classes during the same calendar year (see S1 Text, p. 3). In our main analyses, we excluded individuals who collected single β-blocker prescriptions during follow-up (n=134,336). To address the possibility that these individuals may have stopped taking the medication due to adverse events, we carried out analyses including them. In these analyses, individuals with a single prescription were assumed to be exposed to medication during the three months following their collected prescription. In the main analyses, we also excluded individuals who had been instructed to take the medication as required (PRN) in the prescription text due uncertainty of regular β-blocker use. Because a proportion of these individuals may have been prescribed β-blockers to treat anxiety, we also carried out analyses including them in our main cohort. In these analyses, medication exposure for individuals with PRN instructions was modelled as in our main models (see Medications paragraph). To examine if β-blockers were differentially associated with violent crimes by age, we stratified individuals into different age groups depending on their age during the study period; up to age 30, age 30 to 49, age 50 to 60, and age 70 and older. We then examined associations between β-blockers and violent crime separately for each age group.

As in the abstract, where statistical data are reported, for example line 350 "(HR=1.08, 95% CI=1.02-1.15)" please include p-values
please report data as described previously with the absence of "=" symbol i.e. "(HR: 1.08, 95% CI: 1.02- 1.15, p<0.01)"
where p-values are reported please also include the statistical test used to determine them

Response:
We have added p-values throughout the Results and Discussion, included the statistical test in our Results section, and also removed the "=" symbol.

TABLES

Please re-title table 1 to read "Baseline characteristics of the study cohort" or something similar

Where 95% CIs reported please also include p-values. In the table caption/legend please state the statistical test used to determine them

Response:

We have re-titled table 1 as suggested. We have also included p-values where 95% CIs are reported and stated the statistical test used.

FIGURES

For all figures please include an appropriate figure caption/legend which appropriately describes the data presented in the figures. Please include and define any abbreviations. Please check and amend throughout all figures (and tables) including those in the supplementary files.

Response:

We have included figure captions/legends which describe the data presented and defined abbreviations for figures and tables in the manuscript and in the supplements. We have also improved the graphic design of figures.

DISCUSSION

Please remove the headings "strengths and limitations" and conclusions from below the discussion and structure the discussion as follows: a short, clear summary of the article's findings; what the study adds to existing research and where and why the results may differ from previous research; strengths and limitations of the study; implications and next steps for research, clinical practice, and/or public policy; one-paragraph conclusion.

Response:

We have removed both headings, and also structured the Discussion as suggested. We also added next steps for research and a concluding paragraph (10th and 11th paragraphs of the Discussion):

Our findings demonstrated reduced associations with charges for violent crimes during β-blocker treatment. More studies using other designs (e.g. randomised controlled trials), are needed to better understand the role of β-blockers in the management of aggression and violence.

In conclusion, the use of β-blockers to manage anxiety is not supported in this real-world study of new presentations of anxiety in secondary patient care. If triangulated using other designs, β-blockers could be used to manage aggression and hostility in individuals with psychiatric conditions.

REFERENCES

Please ensure you have followed our guidelines for listing references which can be found here: https://journals.plos.org/plosmedicine/s/submission-guidelines#loc-references
Journal name abbreviations should be those found in the National Center for Biotechnology Information (NCBI) databases.

Response:
We have done this.

SOCIAL MEDIA

In the event that your manuscript is published, to help us extend the reach of your research, please provide any Twitter handle(s) that would be appropriate to tag, including your own, your coauthors', your institution, funder, or lab. Please respond to this email with any handles you wish to be included when we tweet this paper.

Response:
@seenafazel
@OxPsychiatry
@CNS_KI
@RalfKH

Comments from the Academic Editor:

Its a really interesting paper and addresses an important (if untrendy) topic with an

amazing database. The findings have potential to influence future research and practice. They have been careful with their interpretation and rigorous with sensitivity analyses. As long as the statistical analytical concerns are not fatal, it looks as if the reviewer's points can all be addressed. The reviewers were thorough and have articulated most of the issues that I noted. I had one confusion which you might ask the authors to address (unless you can see that they address it and I missed it):

- how were repeated events of the same type of outcome in one individual handled within the analysis?

Response:
Thank you for these positive comments.
As for the specific query, we agree that this would be helpful to clarify in the paper. In the analyses, each event of the same type of outcome was treated as a distinct observation, and the underlying time scale for each individual was reset to time zero when there was an outcome (or event), and a new time period started.

We have clarified this in our Statistical analyses, 2nd paragraph:
<u>In the analyses, the underlying time scale was divided into several periods; each individual was followed from the start of the period (time zero) until treatment switch (i.e. from no treatment to treatment or vice versa), the occurrence of an event (outcome), or they became one year older in age, whichever came first (consequently, each period could be up to 365 days). After this, a new period started, time was reset to zero, and the individual was followed up until treatment switch, event, or next birthday. This was done until the individuals were censored at death, emigration, or the end of study period. Each time-to-event was thus treated as a distinct observation. Because time-at-risk was measured from the start of all periods, recurrent events were accounted for.</u>

In our Methods – Psychiatric and behavioural outcomes, 1st paragraph, we also added:
<u>Each event was treated as a distinct observation, meaning that individuals could experience repeated events of the same outcome. If more than one event of the outcome of interest was registered on the same day (e.g. more than one violent crime), only one event was counted that day.</u>

Comments from the reviewers:

Reviewer #1:

This is an interesting population-based study on the associations between β-blockers and psychiatric and behavioural outcomes. However, there are a few major issues needing attention.

1) Authors said "We applied within-individual Cox proportional hazards regression to compare periods on treatment with periods off treatment in order to reduce possible confounding by indication". Using self controls is an alternative to standard epidemiology method, but not sure and not convinced how to apply this within the Cox model framework. There is no methodogical reference on this within-individual cox model especially statistically.

Response:
Thank you for this helpful comment. We agree that references in support of the methods and some more explanation would improve the paper.

Specifically in relation to the Cox models, within-individual analyses can conveniently be carried out with stratified Cox regression for time-to-event data (see Allison PD. Fixed effects regression models. Thousand Oaks, CA: SAGE Publications, 2009; and also Gunasekara et al., Int J Epidemiol 2014;43:264–269. doi:10.1093/ije/dyt221 [about fixed-effects models in repeated measures data]). Consistent with this, within-individual Cox models have been used in previous large pharmacoepidemiological studies (see e.g. Lichtenstein et al., N Engl J Med 2012; Molero et al., PLoS Med 2015; Taipale et al., Lancet Psychiatry 2022). To explain model choice and clarify methods, in the revision, we describe the stratified Cox regression in more detail. In brief, the possibility to estimate within-individual effects comes from using an underlying time-scale which is reset to 0 at treatment switch, outcome event, or at their birthday. In addition, we adjust for age at the start of each time period. We have also included the Allison and Gunasekara methodological references.

Statistical analyses, 2nd paragraph (underlined added):

Our null hypothesis was that no associations would be demonstrated between β-blockers and psychiatric hospitalisations, suicidal behaviour, and violent crime. A within-individual design – using stratified Cox proportional hazards regression - was applied to examine associations. The reason for using a within-individual design rather than standard between individual design, was that the between-individual design is susceptible to individual-specific unmeasured confounders that affect both the selection into β-blocker treatment and the tested outcomes. The current study design is a form of self-controlled case series design where each individual is entered as a separate stratum in the stratified Cox regression, and periods on medication are compared to periods off medication within the same individual (54). Mathematically, the model is given by

$$\lambda\left(t_{ij}\middle|P_{ij}, X_{ij}, individual\ i\right) = \lambda_{0i}\left(t_{ij}\right)e^{\beta P_{ij}+\gamma X_{ij}}$$

where $\lambda\left(t_{ij}\middle|P_{ij}, X_{ij}, individual\ i\right)$ is the conditional hazard function at time t_{ij}, given P_{ij}, X_{ij} (where P_{ij} is the exposure and X_{ij} the vector of measured covariates) and the individual i. By conditioning on the individual, and assuming individual-specific baseline hazards (the $\lambda_{0i}(t_{ij})$ in the equation), the model implicitly adjusts for all stable (i.e. time-invariant) confounders that are not readily observed in register data (such as genetic and other background risk factors) within the individual; these are absorbed by the individual-specific baseline hazard. This design also allowed us to adjust more fully for confounding by indication that was stable during the study period. In the analyses, the underlying time scale was divided into several periods; each individual was followed from the start of the period (time zero) until treatment switch (i.e. from no treatment to treatment or vice versa), the occurrence of an event (outcome), or they became one year older in age, whichever came first (consequently, each period could be up to 365 days). After this, a new period started, time was reset to zero, and the individual was followed up until treatment switch, event, or next birthday. This was done until the individuals were censored at death, emigration, or the end of study period. Each time-to-event was thus treated as a distinct observation. Because time-at-risk was measured from the start of all periods, recurrent events were accounted for.

The within-individual design does, however, not adjust for time-varying factors, i.e. those that changed during follow-up (e.g. age, health status, or use of other medications). We therefore also adjusted for age as a continuous time-varying covariate at the start of each time period in all our analyses. We also used a quadratic function of age as a time-varying covariate at the start of each time period to allow for non-linear effects in all our analyses. The within-

individual model has been applied in several studies of associations between medications and psychiatric and behavioural outcomes (63, 67), and underlying methods are discussed elsewhere (61, 68).

2) As it's a time to event analysis, if a patient have multiple on and off treatment, how can you define the time? added up? Any washout time between on and off treatment? Any long term effect of Beta blocker after off treatment? But that becomes unnatural and interrupted.

Response:
Our study period is divided into several periods where every treatment switch, every outcome event, and every birthday mark the start of a new period. Individuals are thus followed from the start of each period ('time zero') until treatment switch, the occurrence of an outcome event, or they turn one year older. Consequently, each period can be up to 365 days. After this, a new period starts and time is reset at zero. The underlying time scale is therefore time since each respective period start. We have clarified this in our statistical analyses section (please see text above).

In our main analyses, we did not include a wash-out period between treatment and non-treatment periods as β-blockers are eliminated relatively rapidly from the body (most β-blockers have an elimination half-life between 3 to 10 hours; Helfand H et al., Drug class review on beta adrenergic blockers, 2007). We defined the end of a treatment period as the day of the last dispensed prescription. This interruption of the medication exposure gives a more conservative estimate of the exposure on the outcome, and does not account for late treatment or discontinuation effects of the medication. To test the possibility of these effects, we carried out sensitivity analyses where we modelled individuals as exposed for the three months after their last dispensed prescription (see Sensitivity analyses – alternative exposures and outcomes, 1st paragraph). These results remained similar to the main analyses (Table 2), suggesting no discontinuation effects. We now address this in the Discussion, 8th paragraph: In our primary analyses, we defined the end of a treatment period as the day of the last dispensed prescription, which gives a more conservative estimate of medication exposure. However, our sensitivity analyses accounting for discontinuation or late treatment effects showed no differences in associations.

3) Also how about proportional hazard assumption? Censoring? Cox model was not designed for this type of self control and I would like to see the theoretical proof and justification of applying Cox model in this setting properly.

Response:
We did not test the proportional hazards assumption as it is not crucial to have proportional hazards in order to perform a Cox regression (Stensrud & Hernán. JAMA. 2020) because the hazard ratio is expected to vary over follow-up. The hazards are only proportional if the hazard ratio remains constant from start until the end of the follow-up, which is not the case for most medical interventions. Treatment effects may change over time, and the hazard ratio is therefore used as a convenient summary (i.e. a weighted average of the time-varying hazard ratios) of the treatment effect for the entire follow-up. For a more detailed explanation on why testing the proportional hazards is not essential, see Stensrud & Hernán. JAMA. 2020 14;323:1401-1402. doi: 10.1001/jama.2020.1267. We have added a note on this in the Statistical analyses section, 2nd paragraph:
<u>We did not test for proportional hazards as they were expected to vary over follow-up.</u>

The reason for using the stratified Cox model was that estimates in the regular Cox model (i.e. a between-individual model) may be susceptible to bias from unmeasured confounders that affect both the selection into β-blocker treatment and psychiatric and behavioural outcomes. The stratified Cox model implicitly adjusts for all time-invariant confounders within each individual - whether these confounders were measured or unmeasured - such as genetics, or early family environment, because each individual acts as their own control. We have added a justification for applying the Cox model in this setting, including a theoretical explanation of the model and citations in support, in our response to comment 1.

4) Competing risk. For outcomes other than all cause mortality in survival analyses, competing risk (from death) need to be considered and adjusted using methods such as fine and grey model. However, this issue was not considered at all in the paper, therefore the resulted HRs could be inaccurate and need to be adjusted for competing risk.

Response:

This is an important comment to address, as it provides an opportunity to respond to a common misconception regarding competing risks and the Fine and Gray model. The Fine and Gray model has been suggested as a method for adjusting for competing risks, but our understanding is that the model was not introduced for this purpose, and not valid for causal inference in setting such as ours. In fact, we understand that using the Fine and Gray model to estimate hazard ratios of an event in the presence of competing risks would likely increase confounding rather than remove it. This is because this model estimates the subdistribution hazard, which relates covariates to the cumulative probability of the event of interest in the presence of competing risks. In practice, this means that when an individual experiences a competing risk (e.g. dies from any cause), they are not censored (i.e. removed from the risk set), but remain in the risk set. This becomes “unnatural” since an individual that dies cannot be at risk of experiencing the event of interest in the future. However, keeping individuals who experience the competing event in the risk set is necessary in order to estimate the cumulative incidence. The cumulative incidence is useful for prediction, e.g. if you have a cohort of newly diagnosed cancer patients, and you want to predict how many will have died of cancer and how many have died of other causes at the end of a defined time period. However, it is not useful for analysing the causal effects of competing risks. In fact, it confounds them; a variable that increases event A will decrease event B, simply because event A will eliminate the possibility of event B.

To give an example: In our cohort, the Fine and Gray model would estimate the cumulative probability of suicidal behaviour (i.e. the event of interest) in the presence of death by other causes (i.e. a competing risk). However, the effect estimate for β-blockers on suicidal behaviour could be driven (entirely or partly) by the effects of β-blockers on death by other causes. This would mean that, even in the complete absence of any direct effect of β-blockers on suicidal behaviour, the Fine and Gray model would estimate an increased subdistribution hazard ratio if β-blocker users were more likely to die from suicide simply as a result of being less likely to die from other causes (that is, because β-blockers are used to treat e.g. cardiac conditions, and thereby reduce the risk of dying). We therefore believe that it is not useful to apply the Fine and Gray model. We have motivated this is the Statistical Analyses section and, more detailed, in the Supplemental Material (see below). For more information and an example, please see Bhaskaran et al., Eur Urol 2013:64; e86-e87 https://doi.org/10.1016/j.eururo.2013.07.004 (which is a commentary on Grytli et al., Eur

Urol 2014;65:635-41. doi: 10.1016/j.eururo.2013.01.007. where the researchers incorrectly had used the Fine and Gray model to estimate the effect of β-blockers on prostate cancer).

In the Statistical analyses, 2nd paragraph, we added:
To estimate cause-specific hazard ratios, we treated the competing event of death as a censoring event, rather than fitting competing risks models (see S1 Text p. 6 for more details).

In the Supplemental Material, p. 5, we added:
To estimate cause-specific hazard ratios, we treated the competing event of death as a censoring event, rather than fitting competing risks models. Using a competing risk analysis, such as the Fine and Gray model, would estimate the subdistribution hazard ratio. Such a hazard ratio would not only capture any potential effect of exposure (β-blockers) on the outcome of interest (psychiatric hospitalisations, suicidal behavior, or violent crime) but would also be influenced by any potential effect of the exposure on the competing event (death) [19]. In the current study, this was not appropriate, wherefore we opted to estimate the cause-specific hazard ratio.

5) Quite a few writing in the paper is difficult to follow and confusing, such as in the findings in the abstract: "There was heterogeneity in the direction of results; there were reductions in psychiatric hospitalisations...". Then, what compares what? Normally we would say "treatment A, comparing to treatment B, reduced the risk of hospitalisation by x%". There are many places in the paper where the interpretation of HRs needs to improve.

Response:
Thank you – we agree and done through the paper with this in mind. In the Abstract, we now state:
There was heterogeneity in the direction of results; within-individual analyses showed that periods of β-blocker treatment were associated with reduced hazards of psychiatric hospitalisations (hazard ratio [HR]: 0.92, 95% confidence interval [CI]: 0.91-0.93, p<.001) and charges of violent crime (HR: 0.87, 95% CI: 0.81-0.93, p<.001), and increased hazards of suicidal behaviour (HR: 1.08, 95% CI: 1.02-1.15, p=.012).

We also clarified the findings in the Results 3rd paragraph (underlined added):

We carried out analyses comparing all treatment periods to all non-treatment periods within each individual using stratified Cox proportional hazards regression (Fig 1; event rates in Table 1). Results from our within-individual analyses showed that periods on β-blocker treatment were associated with a lower hazard ratio of psychiatric hospitalisations (hazard ratio [HR]=0.92, 95% confidence interval [CI]=0.91-0.93, p<.001). We found increased hazards of suicidal behaviour during β-blocker treatment periods (HR: 1.08, 95% CI: 1.02-1.15, p=.013), and reduced hazards of violent crime (HR: 0.87, 95% CI: 0.81-0.93, p<.001).

We have made similar clarifications in the Results and Discussion.

Reviewer #2:
This is an interesting longitudinal study using linked registry data that examines the associations between beta blockers and psychiatric events in Sweden. The study and it's findings suggest that beta blockers contribute to or are associated with increases in suicidal risks but not anxiety or violent crimes.

I have several comments that warrant some revisions to strengthen the paper.
1) It is not clear why authors report psychiatric outcomes in aggregate. It is clear from prior literature that beta blockers are associated with suicidal symptom or depression but less on anxiety and criminal behavior. This study reinforces this given heterogeneity in effects.

Response:
We were perhaps not entirely clear about this – we did not include suicidal outcomes in our measure of psychiatric hospitalizations (and have clarified this in the Abstract and Methods now). But the reviewer is right insofar as psychiatric hospitalizations does include any admissions for any psychiatric diagnosis. The main reason for this is that psychiatric problems are often co-morbid, particularly severe psychiatric disorders (i.e. that lead to a hospitalisation). At the same time, we agree with the reviewer that using an aggregate measure could mask heterogenous associations between β-blockers and different diagnoses. We therefore carried out sensitivity analyses where we examined hospitalisations due to either depressive, anxiety, or psychotic disorders separately (Table 2), since these diagnoses have been linked to β-blocker use in previous studies. In these analyses we found reduced hazards

for hospitalisations due to depressive and psychotic disorders during β-blocker treatment, and no associations with hospitalisations for anxiety disorders. This suggests that observed reductions in psychiatric hospitalisations may be driven by the reductions in depressive and psychotic disorders (discussed in paragraph 3 of Discussion). We agree that this could be clarified further, and have added the following to the Abstract (underlined added):
There was heterogeneity in the direction of results; within-individual analyses showed that periods of β-blocker treatment were associated with reduced hazards of psychiatric hospitalisations (hazard ratio [HR]: 0.92, 95% confidence interval [CI]: 0.91-0.93, p<.001) and charges of violent crime (HR: 0.87, 95% CI: 0.81-0.93, p<.001), and increased hazards of suicidal behaviour (HR: 1.08, 95% CI: 1.02-1.15, p=.012). After stratifying by diagnosis, reduced associations with psychiatric hospitalisations during β-blocker treatment were mainly driven by lower hospitalisation rates due to depressive (HR: 0.92, 95% CI: 0.89-0.96, p<.001) and psychotic disorders (HR: 0.89, 95% CI: 0.85-0.93, p<.001). Reduced associations with violent charges remained in most sensitivity analyses, while associations with psychiatric hospitalisations and suicidal behaviour were inconsistent.

We also clarified that we did not include suicidal outcomes in our measure of psychiatric hospitalizations in the Abstract (underlined added):
We linked register data on dispensed β-blocker prescriptions with main outcomes; hospitalisations for psychiatric disorders (not including self-injurious behaviour or suicide attempts), suicidal behaviour (including deaths from suicide), and charges of violent crime.

And in the Methods (underlined added):
Outcomes included: 1) hospitalisations due to a psychiatric disorder (International Classification of Diseases, 10th revision [ICD-10]: F10-F69, F80-F99, excluding organic and intellectual disability disorders, and self-injurious behaviour or suicide attempts)

2) The rationale for the use of antihistamines (vs other meds)as a control is not clear and potentially problematic l. First antihistamines is a therepautic category with many very different drugs. Second, antihistamines—many of them- are available over the counter and not clear if the medication data captures over the counter dispensings or sales. Third, why not use an antihypertensive and limit to older age groups? For example ace inhibitors?

Response:
This is a very helpful suggestion, which we consulted about with clinical colleagues – and we agree. So, we have added sensitivity analyses using ACE inhibitors as a negative control medication. In these analyses, ACE inhibitors were used as an independent exposure among those who had been treated with this medication in the β-blocker cohort (n=561,868). Because ACE inhibitors may be co-prescribed with β-blockers, we also adjusted for the concurrent use of β-blockers in these analyses. Our results on ACE inhibitors (Table 2) are broadly reassuring – and find no non-specific treatment effects for psychiatric and violent outcomes, and a small (possibly chance) increased risk of suicidal events, which suggests that associations are not specific for β-blockers.

We have added the following to the Methods, sensitivity analyses – posthoc analyses, 1st paragraph:
We further examined non-specific treatment effects by using a different negative control medication - angiotensin-converting-enzyme (ACE) inhibitors (ATC: C09AA) - as an independent exposure in the β-blocker cohort (see S1 Text p. 4 for details).

Supplementary material, Negative controls, 1st paragraph (underlined added):
We used two medications - angiotensin-converting-enzyme (ACE) inhibitors (ATC: C09AA) and antihistamines for systemic use (ATC: R06A; excluding phenothiazine derivatives [ATC: R06AD], as these are used clinically as mild sedatives to treat anxiety) - as negative controls to test for non-specific treatment effects. This choice was determined on the basis of theoretical reasons; ACE inhibitors are prescribed for similar indications and in similar settings as β-blockers, and would thus likely capture similar non-specific treatment effects, such as increased supervision and healthcare contacts. Antihistamines, on the other hand, are prescribed for other indications and in other settings than β-blockers, and would thus likely capture non-specific treatment effects not related to cardiac treatment (since β-blockers can be prescribed for non-cardiac indications). In these analyses, the negative control medications were used as an independent exposure in the β-blockers cohort, and treatment periods were defined in the same manner as β-blocker treatment periods (i.e. at least two dispenses within six months). Because ACE inhibitors and β-blockers are often co-prescribed, we adjusted for the effect of concurrent β-blocker use in these analyses. Information was extracted from the Swedish Prescribed Drug Register.

Results, sensitivity analyses – alternative exposures and outcomes, 2nd paragraph:
We also repeated our main models using two negative controls – antihistamines and ACE inhibitors - as independent exposures in the β-blockers cohort to examine non-specific treatment effects. Results showed no associations with psychiatric hospitalisations (HR=1.00, 95% CI=0.95-1.05), suicidal behaviour (HR=1.16, 95% CI=0.99-1.36) or violent crime (HR=1.23, 95% CI=0.93-1.63) during antihistamine treatment periods, and increased hazards of suicidal behaviour (HR: 1.15, 95% CI: 1.02-1.31, p=.020), and no associations with psychiatric hospitalisations (HR: 0.99, 95% CI: 0.96-1.01, p=.208) or violent crime (HR: 1.01, 95% CI: 0.91-1.14, p=.813) during ACE inhibitor treatment periods.

Discussion, 6th paragraph:
We also examined if associations could be attributed to non-specific treatment effects, such as increased supervision or healthcare contacts, by using another cardiac medication (ACE inhibitors) and a non-cardiac medication (antihistamines) as independent exposures in the β-blockers cohort. In these analyses, we found no clear associations with psychiatric hospitalisations or violent crime, and small increased links with suicidal behaviour. If associations were to be confounded by non-specific treatment effects, we would have expected similar patterns for all outcomes during treatment with ACE inhibitors and antihistamines, as during β-blocker treatment. The differing treatment patterns for psychiatric hospitalisations and violent crime suggest that non-specific treatment effects were not prominent. The increased links with suicidal behaviour could suggest that associations were not specific (i.e. causally related) to β-blockers.

3) Not clear how analyses accounted for the initiation or dispensing of antidepressants. This needs to clarified and addressed.

Response:
Agree that this needs clarification. We have added the following to the Sensitivity analyses – alternative samples, 1st paragraph (underline added):
Because β-blockers combined with selective serotonin-reuptake inhibitors (SSRIs) have been linked to reduced depression (33, 72, 73), we addressed the confounding effects of antidepressant use in sensitivity analyses. In these analyses, we excluded all individuals who had collected an antidepressant (i.e. an SSRI or another antidepressant, ATC: N06A) during the study period (i.e. 2006-2013) from the cohort, and examined associations between β-

blockers and outcomes in those who remained (i.e. those who had not collected an antidepressant during the study period). We also carried out analyses where we excluded individuals who had collected an antipsychotic medication (ATC: N05A), or common hypertension medications including calcium channel blockers (ATC: C08), renin-angiotensin system acting agents (ATC: C09), or statins (ATC: C10AA), respectively, to address confounding effects by other medications on psychiatric and behavioural outcomes.

Reviewer #3:
Thank you for the opportunity to review this important paper. I did not review the original version, so my comments are from a 'first read' perspective. You have carefully presented a series of complex and well-thought out SCCS, and epidemiologists (and hopefully clinicians!) will enjoy reading this from both the clinical-implications and methodological perspectives. You have answered most of the questions I had as the manuscript went on, and present a balanced conclusion taking all analyses into account.

Comments for your consideration:
1) The introduction provides a sound overview of the literature. The only missing comment might be about adverse drug reaction labelling of b-blockers-as some psychiatric events and sleep disorders are listed in SPC as potential ADRs.

Response:
This is a good point. We have added the following to the Introduction, 1st paragraph (underlined added):
However, there have been concerns of psychiatric adverse events during β-blocker use (5), and sleep disturbances, psychoses, and depression are listed as potential adverse events in the summary of product characteristics for β-blockers (6).

2) I understand that you have included information about registries in the supplementary material, but a brief comment about linked data sources in the design, will also be helpful.

Response:
We agree, and have added this to the Methods – design, 1st paragraph (underlined added):

We conducted a population-based longitudinal cohort study using Swedish nationwide registers linked through each person's unique identification number (55). Registers included the Total Population Register (for information on age, sex, and migration), the Swedish Prescribed Drug Register (for information on dispensed medications), the Swedish Patient Register (for information on diagnoses, hospitalisations, and treatment of suicidal behaviour), the Cause of Death Register (for information on death by suicide and other causes), the Register of Persons Suspected of Offences (for information on charges for violent and non-violent crime), the Longitudinal Integrated Database for Health Insurance and Labour Market Studies (LISA; for information on civil status and source of income), and the Prison and Probation Services Register (for information on periods in prison) (55-60). For more details on the registers, see S1 Text, p. 2. We applied a within-individual design (61) that inherently adjusts for all stable confounders, i.e. factors that do not change during the study period (e.g. genetics and health history), and more fully adjusts for stable factors associated with confounding by indication.

We also added the following to the Supplementary Methods – Registers, 1st paragraph: Registers included the Total Population Register, the Swedish Prescribed Drug Register, the Swedish Patient Register, the Cause of Death Register, the Register of Persons Suspected of Offences, and the Prison and Probation Services Register [1-7]. The Total Population Register contains information on birth, death, sex, and migration for the entire Swedish Population. The Swedish Prescribed Drug Register includes information on all prescriptions that are dispensed from all pharmacies in Sweden, and has less than 0.3% missing information. The Swedish Patient Register includes all admissions to all hospitals, as well as all outpatient contacts with specialized secondary care in Sweden. The primary diagnosis is listed in 99% of all hospital discharges, and the positive predictive value of medical diagnoses in this register is around 85-95%. In a previous register-based study, depression diagnoses were validated by comparing concordance rates with another clinical register that was based on multidisciplinary inpatient assessments, to make diagnoses as a gold standard. Results showed fair to moderate agreement (κ of 0.32; 88% full agreement) [8]. Missing data in The Swedish Patient Register is around 1% for inpatient treatment, and around 3% for outpatient treatment. The Cause of Death Register is a register of all deaths in Sweden, where the underlying cause is specified in 96% of the cases. The Register of Persons Suspected of Offences includes all individuals who are charged with a crime after a completed investigation by police, prosecution services, or customs authority. The Longitudinal Integrated Database for Health

Insurance and Labour Market Studies (LISA) covers the adult Swedish population aged ≥ 16 years and contains sociodemographic data such as family, employment, benefits, and education for each calendar year. The Prison and Probation Services Register includes information on all prison sentences, including the start and end date of each prison sentences.

3) A stronger justification as to why two dispenses were required for inclusion in the cohort will be beneficial. There is potential for survival bias if individuals stopped taking beta-blocker after first use, due to adverse events, and the exclusion of these individuals might direct associations towards the null.

Response:
This is a thoughtful comment. We have added a stronger justification for the requirement of two dispenses for cohort inclusion. To address the potential for survival bias, we also added sensitivity analyses where we included individuals with single dispenses in our cohort. Our results (Table 3) remained similar to the main results.

Methods, medications, 1st paragraph:
Individuals who collected a single prescription may or may not have taken the medication. To address uncertainty over medication adherence, we excluded them from our primary analyses. However, this could potentially increase the risk of survival bias (i.e. that individuals who collected a single β-blocker prescription may have stopped taking the medication due to adverse events, while those who collected several β-blocker prescriptions had fewer adverse events and thus continued taking the medication), and direct associations towards the null. We therefore carried out sensitivity analyses where we included those who had collected a single prescription.

Statistical analyses – posthoc analyses, 1st paragraph:
In our main analyses, we excluded individuals who collected single β-blocker prescriptions during follow-up (n=134,336). To address the possibility that these individuals may have stopped taking the medication due to adverse events, we carried out analyses including them. In these analyses, individuals with a single prescription were assumed to be exposed to medication during the three months following their collected prescription.

Results section, sensitivity analyses - alternative samples, 1st paragraph:

To address a potential for survivor bias in our β-blocker cohort (i.e. that individuals who experienced adverse events discontinued with β-blockers), we carried out analyses where we included individuals who had collected only one prescription in the main β-blocker cohort. Results remained similar to the main analyses.

4) Ending exposure date on prescription date will be an underestimate of exposure, and may introduce potential for misclassification. I am not sure whether all readers would agree with this choice of definition, but you do address this and provide assurance via sensitivity analyses.

Response:
Yes, our sensitivity analyses (Table 3) show that results remain similar when adding the exposure date three months after the collected prescription.

5) Further justification of excluding prn use is required given as this is likely to exclude people prescribed propranolol for prn use for anxiety.

Response:

Another thoughtful comment. We added a justification for excluding individuals with PRN use. We also added sensitivity analyses where we include individuals with PRN instructions, and results (Table 3) were similar to the main results.

Methods – Medications, 1st paragraph:
Furthermore, we had excluded individuals with the instructions in the prescription text to take the medications 'pro re nata' (PRN; i.e. as required) from our cohort due to uncertainty over regular medication use. However, this could increase the risk of selection bias, as a proportion of these individuals may have been prescribed β-blockers to treat anxiety. We therefore carried sensitivity analyses including them.

Methods, sensitivity analyses – posthoc analyses, 1st paragraph:
In the main analyses, we also excluded individuals who had been instructed to take the medication as required (PRN) in the prescription text due uncertainty of regular β-blocker use. Because a proportion of these individuals may have been prescribed β-blockers to treat

anxiety, we also carried out analyses including them in our main cohort. In these analyses, medication exposure for individuals with PRN instructions was modelled as in our main models (see Medications paragraph). To examine if β-blockers were differentially associated with violent crimes by age, we stratified individuals into different age groups depending on their age during the study period; up to age 30, age 30 to 49, age 50 to 60, and age 70 and older. We then examined associations between β-blockers and violent crime separately for each age group.

Results, sensitivity analyses, alternative samples, 1st paragraph:
In our main analyses, we had excluded individuals who had been instructed the medication PRN due to uncertainty of daily use. We carried out sensitivity analyses including them in the main β-blocker cohort, and results were similar (Table 3).

6) My understanding is that there is no primary care diagnostic data, unless this is captured on prescription direction in Sweden (this rarely happens in the UK). How confident can you be of diagnostic classification? A comment about the lack of primary care exposure diagnoses and outcome data (only severe outcomes reported-hospitalisation and death) would be beneficial, including the direction of effect on estimates.

Response:
The reviewer is quite right – the data are secondary care, which has the benefit of specificity and reliability but lacks information on those prescribed beta-blockers in primary care. We are confident of diagnostic validity based on many studies examining diagnoses using the patient register in Sweden. For example, the PPV for cardiac conditions ranges between 81.7 (for heart failure) to 100 (for myocardial infarction). Coverage is also high; primary diagnoses are missing in 0.8% of somatic care and 3.1% of psychiatric care (Ludvigsson JF et al. BMC Public Health. 2011;11:450. doi: 10.1186/1471-2458-11-450). Using secondary care and mortality outcomes will capture more severe cases, although it also has the advantage of capturing outcomes that are more important for healthcare services as they consume more resources. If primary care data were included in our outcome measures (which probably include less severe cases, and where psychiatric diagnoses have been made by primary care physicians rather than by specialists in psychiatry), we would likely get an effect estimate closer to 1.

We have added this as a limitation in the Discussion, 8th paragraph (underlined added):
The use of official registers involves selection effects and will underestimate rates of underlying disorders and outcomes. Using secondary care and mortality outcomes will selectively include more severe cases of disorders, thus our results may not generalise to less severe cases and/or cases that were not diagnosed by specialists in psychiatry. On the other hand, official registers capture information on actual health-care contacts, reflecting real-world outcomes that consume resources.

7) Ethical approval is 9 years old-I presume that you have pan-database approval and this is why the approvals are old. If this is the case, please state this along with how, and when, this individual study was approved.

Response:
We have pan-database approval – and the approvals were given at the time when all the databases were linked and were intended to cover future use of them.

8) Despite your justification in S1, I am unsure antihistamines were the most appropriate choice of negative control, due to seasonal and ad hoc use, and possible misclassification to non-prescription self medication. That said, you have included plenty of thoughtful sensitivity analyses.

Response:
Reviewer #2 also pointed this out, and we have therefore added another negative control - ACE inhibitors - as suggested by reviewer #2. Please see our response to reviewer #2, comment 2 for more details.

9) A few times, you discuss the potential involvement of suicidality following major cardiac events. Perhaps a comment about the psychological burden of sudden and major, life-changing health conditions is warranted, which might be an important factor accounting for any association.

Response:
This is another thoughtful point. We added the following to the Discussion, 4th paragraph (underlined added):
Several psychological reactions are reported to occur after a cardiac event that can affect mood (81); individuals may have negative thoughts about their overall well-being, be uncertain about the future, concerned about reduced physical ability, or feel guilty about previous habits that may have increased the risk of the cardiac event. In line with this, research shows that the risk of suicide is increased during the first months after a cardiac event (53, 82), and one explanation for our findings could be that the psychological burden associated with the cardiac condition, rather than the β-blocker treatment, increases suicidal risk.

Reviewer #4:
Reviewer's comments
Manuscript PMEDICINE-D-22-02228R1 describes an very nice observational study using large cohorts from Swedish national registries investigating β-blockers and psychiatric disorders, suicidal behaviour and violent crime.. Although highly interesting some questions arise:

1) The following registers were consulted: the Total Population Register, the Longitudinal integrated database for health insurance and 214 labour market studies (LISA), the National patient register, the register of people Suspected of Offences and the Swedish prescribed drug register.
These are not described in the text, and also not in the Supplement text as stated. It is strongly advised to briefly describe these registers in a few sentences in the text.

Response:
We have added more information about the registers. Please see our response to Reviewer #3, comment 2.

2) How were 'suicidal behaviour' and 'violent crime assessed'? Only as yes/no (it seems) or with questionnaires? The results should be presented in the manuscript. Now it is only represented as number of events, but this should be explained in depth.

Response:
Both outcomes were assessed through register data, specifically ICD-10 codes (for suicidal behaviour) and charges of violent crimes by police or prosecution services.

We have explained these measures in more detail in our Methods – Psychiatric and behavioural outcomes, 1st paragraph (underlined added):
2) death from suicide, or unplanned (i.e. without prior appointment or referral) hospital and specialised outpatient care visits due to self-injurious behaviour or suicide attempt as registered in mortality or patient records (ICD-10: X60-X84), and; 3) charges of violent crime (i.e. crimes against people in the Swedish penal code) after a completed investigation by police, prosecution service, or customs authority. We used the incident date of the violent crime (i.e. the date when the crime was committed) rather than the date of the charge. Each event was treated as a distinct observation, meaning that individuals could experience repeated events of the same outcome. If more than one event of the outcome of interest was registered on the same day (e.g. more than one violent crime), only one event was counted that day. Data were collected from the National Patient Register (outcomes 1-2) (57), the Cause of Death Register (outcome 2) (56), and the Register of People Suspected of Offences (outcome 3) (60). For more details on outcomes, see S1 Text, p. 3.

In the Results – Characteristics of the β-blocker cohort, 1st paragraph, we added:
During the study period, 6.9% (n=96,801) of the β-blocker users were hospitalised for a psychiatric disorder, 0.7% (n=9,960) presented with suicidal behaviour (i.e. treatment at hospital or specialised outpatient care for self-injurious acts or suicide attempts, or deaths from suicide as the stated cause of death), and 0.7% (n=9,405) were charged with a violent crime (i.e. attempted, completed, and aggravated forms of murder, manslaughter, unlawful threats, harassment, robbery, arson, assault, assault on an official, kidnapping, stalking, coercion, and sexual offences) after a completed investigation by police, prosecution service, or customs authority.

We also clarified this in the Discussion, 1st paragraph:
In addition, there was a 13% (HR: 0.87, 95% CI: 0.81-0.93, p<.001) lower risk of being charged with a violent crime by the police or prosecution services during β-blocker treatment.

3) The association of b-blocker treatment and reduction in violent crime puzzles me. The

total patient group consisted of mostly older people, (>40% over 70 years) and lots of retired people. It could be speculated that the people with crime pasts were less violent because they aged. Although a sensitivity analysis of this special subgroup was performed, it is recommended to also look into this and describe this.

Response:
The reviewer makes a valid point that individuals with a history of violence may be less violent because they aged. However, we adjust for within-individual aging during the study period by including the individual's age at the start of each respective period as a time-varying covariate (please see our response to Reviewer #1, comment 1, for more information on this).

However, we agree with the reviewer that age may be an important factor related to violent crime that warrants further examination. We have therefore carried out sensitivity analyses where we examine if the associations between β-blockers and outcomes differ by different age groups. In these analyses we have stratified individuals into separate age groups depending on their age during the study period (under 30, 30-49, 50-69, and 70+). We also adjusted further for each individual's aging during the study period (i.e. by including age as a time-varying covariate) in the stratified analyses. Our results (Table 3) show that all age groups were associated with decreased hazards of violent crime, although associations in the two youngest groups did not reach statistical significance.

We have added the following to the Methods, sensitivity analyses – posthoc analyses, 1st paragraph:
To examine if β-blockers were differentially associated with violent crimes by age, we stratified individuals into different age groups depending on their age during the study period; up to age 30, age 30 to 49, age 50 to 60, and age 70 and older. We then examined associations between β-blockers and violent crime separately for each age group.
Results, sensitivity analyses – alternative exposures, 3rd paragraph:
Second, we stratified associations by different age groups; up to age 30, 30 to 49, 50 to 60, and 70 and older. We found reduced hazards of violent crime for all age groups during β-blocker treatment periods, although hazards did not reach statistical significance for the two younger groups (HR: 0.78, 95% CI: 0.59-1.03, p=.077; HR: 0.76, 95% CI: 0.56-1.03, p=.073).

Discussion 2nd paragraph (underlined added):
We found the reduced associations with violent crime charges during β-blocker treatment were consistent using alternative time periods, excluding individuals with co-prescribed medications, excluding prevalent users, stratifying by different age groups, and stratifying on hospitalisations for cardiac conditions.

4) Another point is that although some other co-medications were assessed, also benzodiazepines, which could result is less violence, should be assessed. Both of these should be addressed in in the manuscript.

Response:
This is a helpful suggestion. Because benziodiazepines may be associated with both psychiatric and violent outcomes, we have carried out new sensitivity analyses where we exclude individuals who were prescribed benzodiazepines during follow-up and examined all outcomes. Our results (Table 3) show similarly reduced associations with psychiatric hospitalisations and violent crimes (although these did not reach statistical significance), and no clear associations with suicidal behaviour.

We have added this to our Methods, sensitivity analyses – posthoc analyses, 1st paragraph:
Furthermore, we carried out analyses where we excluded individuals who had been prescribed benzodiazepines (ATC: N03AE, N05BA, N05CD, N05CF) to address the confounding effects of concurrent benzodiazepine use on psychiatric and behavioural outcomes.

Results:
To account for potentially confounding effects by other medications (Table 3), we carried out analyses excluding individuals prescribed psychotropic (i.e. antidepressants or benzodiazepines) or cardiac medications (i.e. calcium channel blockers, renin-angiotensin system acting agents, or statins), and individuals with polypharmacy (i.e. five or more different medication classes during the same calendar year). Associations remained similar to the main analyses when excluding individuals with each respective medication or polypharmacy.

5) When investigating drug effects, it is always important to assess co-medication and

especially polypharmacy, as interactions may occur that influence behavior. Was this assessed? It is shortly mentioned but given it's potential importance as a confounder results should be shown and it should be described in more detail in the manuscript.

Response:
Another good suggestion. Adjusting for polypharmacy is important since interactions between medication could affect outcomes. We have therefore carried out sensitivity analyses where we identified all individuals with five or more different medication classes during the same calendar year (we chose five classes since this is the most common definition of polypharmacy – see Masnoon, BMC Geriatr 2017:17:230 https://doi.org/10.1186/s12877-017-0621-2). To ensure that polypharmacy did not affect associations with outcomes (either during β-blocker treatment periods or non-treatment periods), we then excluded the individuals with polypharmacy from the cohort and carried out a new set of within-individual analyses (i.e. including only those without polypharmacy). Our results showed similar associations with psychiatric and violent crime outcomes, and increased associations with suicidal behaviour were no longer statistically significant (Table 3):

We have added this to the Methods, Sensitivity analyses – posthoc analyses, 1st paragraph:
We also controlled for the confounding effects of polypharmacy by excluding individuals who had been prescribed five or more different medication classes during the same calendar year (see S1 Text, p. 3).

Supplemental material p. 5:
In further sensitivity analyses, we excluded individuals with polypharmacy, that is, those who had collected prescriptions for five or more different medication classes, including β-blockers, during the same calendar year. Medication classes included medications for the alimentary tract and metabolism (ATC: A01, A02, A07, A10), cardiovascular medications (ATC: C01-C03, C05, C07-C10), dermatologicals (ATC: D04-D07, D11), genito-urinary system and sex hormones (ATC: G02-G04), systemic hormonal preparations (ATC: H01-H05), anti-infectives (ATC: J01, J02, J04, J05), antineoplastic and immunomodulating agents (ATC: L01-L04), musculo-skeletal system medications (ATC: M01), psychotropics (ATC: N), anti-parasitics (ATC: P01, P02), respiratory system agents (ATC: R01, R03, R05, R06), and sensory organ medications (ATC: S01).

Results, sensitivity analyses – alternative samples, 1st paragraph (underlined added):

To account for potentially confounding effects by other medications (Table 3), we carried out analyses excluding individuals prescribed psychotropic (i.e. antidepressants or benzodiazepines) or cardiac medications (i.e. calcium channel blockers, renin-angiotensin system acting agents, or statins), and individuals with polypharmacy (i.e. five or more different medication classes during the same calendar year). Associations remained similar to the main analyses when excluding individuals with each respective medication or polypharmacy.
