# Supplementary material for: Associations between β-blockers and psychiatric and behavioural outcomes: A population-based cohort study of 1.4 million individuals in Sweden
Source: PLoS Med. 2023 Jan 31;20(1):e1004164. doi: 10.1371/journal.pmed.1004164 (PMC9888684; doi:10.1371/journal.pmed.1004164)
Supplement: Attachment — Submitted filename: Editorial comments_Molero_PLoSMed_REVISION2.pdf [file pmed.1004164.s010.pdf]

Requests from Editors:

GENERAL
1)
Thank you for your exceptionally detailed and considerate response to editor and reviewer requests. Please see below for further minor changes which we request that you address in full.
We note the reviewer comment regarding language and clarity and have some suggestions below, otherwise our copyediting team will help with minor grammatical revisions, as necessary.

Response:
Thank you for these positive comments, and for the suggestions on how to improve the our manuscript. We have addressed each of the editorial and production comments below.

2)
REQUEST TO CHANGE AUTHORSHIP
We accept the request to add the author to the author list given the described contribution made. We thank you for including the letter signed by all co-authors which demonstrates that they consent to the change in authorship.

Response:
Thank you.

3)
ABSTRACT
Thank you for including p-values and revising the presentation of your statistical reporting please report p as <0.001 or 0.012, as opposed to <.001. Please check and mend throughout all sections of the manuscript.

Response:
We have checked and mended the p-values throughout all sections of the manuscript, in the tables, and in the appendix.

4)
AUTHOR SUMMARY
Line 95: "treated with β-blockers using a within-individual design…" it may be apparent to the general reader what a within individual design refers to. Would perhaps benefit from brief clarification or use of a more "lay" term (you do this very nicely in the introduction at lines 178-179)

Response:
A good suggestion. We added the explanation from lines 178-179 in the introduction to clarify for readers (underlined added):
We examined a population-based cohort of 1,400,766 persons in Sweden who had been treated with β-blockers using a within-individual design<u>; that is, we compared individuals to</u>

themselves during medication and non-medication periods to account for background factors that may confound associations.

5)
Similarly, line 108: “If findings on violence are triangulated using other designs…” what does it mean to triangulate findings?

Response:
We agree that this might be unclear, and have made the following clarification:
If findings on violence are confirmed by studies that use other designs, β-blockers could be considered to manage aggression and hostility in individuals with psychiatric conditions.

6)
METHODS and RESULTS
Line 194: 2…on the registers, see S1 Text, p. 2.” When referring to the supplementary data please use “page” instead of “p.” – please check and amend throughout.

Response:
We have checked and changed ‘p.’ to ‘page’ throughout.

7)
Line 463 onwards: please report p-values as <0.001 not .001
TABLES and FIGURES
Throughout, in all tables and, as above, please report p as <0.001

Response:
We now report the p-values in the suggested manner in all sections of the manuscript, in all tables, and in the appendix.

8)
Table 1: Under the characteristic “sex” this should read female and male as opposed to women and men

Response:
We have changed to female and male in Table 1.

9)
For the purposes of transparent data reporting, PLOS Medicine requests that where adjusted analyses are presented, unadjusted analyses are reported concurrently.
For figures 1, 2, 3 & S2 and tables 2 & 3 please indicate whether your analyses are adjusted and if so, please detail in the table/figure caption or footnote which factors are adjusted for.

Response:
All our analyses were adjusted for age (in addition to the already specified specific adjustments in the sensitivity analyses). We have clarified this in the table/figure captions of figures 1, 2, 3 & S2 and tables 2 & 3 by adding:

Age-adjusted within-individual associations…

10)
Please also include the unadjusted analyses for comparison.
Please ensure that p-values are reported (presented as described above) alongside 95% CIs.

Response:
For all analyses in figures 1, 2, 3 & S2 and tables 2 & 3, we now include unadjusted analyses, and present them in S4 Table. In S4 Table, 95% CIs are reported and p-values are presented as suggested above.

11)
Figure 1,2,3 & S2: please indicate in the figure caption/footnote what the dots and lines represent

Response:
We have added the following footnote (new text underlined) to Figures 1,2,3 & S2:
HR = Hazard ratio (represented by the dot); CI = Confidence interval (represented by the line)

12)
REFERENCES
Throughout, please ensure that citations are placed in square brackets
Line 349: "…phase following a cardiac event (69, 70)…" please remove spaces between citations as follows: [69,70]
Please check and amend throughout

Response:
Spaces have been removed and citations have been placed in square brackets throughout the manuscript and in the appendix.

13)
SOCIAL MEDIA
Please include your twitter handles in the manuscript submission form (if not already done so)

Response:
We have included them now.
